# Retraction: MiR-130a-3p inhibits the viability, proliferation, invasion, and cell cycle and promotes apoptosis of nasopharyngeal carcinoma cells by suppressing BACH2 expression

**DOI:** 10.1042/BSR-2016-0576_RET

**Published:** 2023-03-09

**Keywords:** BACH2, miR-130a-3p, nasopharyngeal carcinoma

This article is being retracted from *Bioscience Reports* at the request of the authors following receipt of a notification from a reader, alerting the Editorial Office to multiple concerns within the paper. Various images seem to appear in papers by other, unrelated authors. The Figure 2C control and miR-NC panels and 5C control panel seem to appear in Figure 3B from Feng et al. 2017 (doi: 10.1042/BSR20170019), Figure 3B from Guan et al. 2018 (doi: 10.1111/jcmm.13620) and in Figures 3A, 3C and 7D from Li et al. 2017 (doi: 10.18632/oncotarget.22133). The GAPDH Western Blots from Figure 1D also seem to appear as the Figure 1D GAPDH blots from Yin et al. 2018 (doi: 10.1111/jcmm.13888). Multiple duplications have also been identified within the paper by both the Reader and the Editorial Office between features of the Figures 3 and 6 panels, which include the following:

- Figure 3A, 48h/control and 48h/miR-NC panels
- Figure 3A 0h/miR-mimics and Figure 6A 24h/siRNA-NC panels
- Figure 6A 48h/control and 48h/siRNA/NC
- Figure 3A 48h/control and Figure 6 48h/control
- Figure 3A 48h/miR-NC and Figure 6 48h/siRNA-NC

It has also been noted that the middle scatter plot graphs of Figures 2E and 5E (the FITC miRNA-NC and FITC siRNA-NC plots respectively) seem to share heavily similar features. It has also been noted that the Figure 6E E-cadherin and Vimentin blots seem to be duplicates after a 180-degree rotation. The authors are unable to correct the article and wish to retract the article. The Editor-in-Chief and Editorial Board agree with the retraction.

